# New insights into the role of metallothioneins in obesity and diabetes

**DOI:** 10.1038/s41366-025-01850-1

**Published:** 2025-07-14

**Authors:** Ana Maria Jimenez Jimenez, Hana Michalkova, Sona Krizkova, Moacyr Jesus Barreto de Melo Rego, Vojtech Adam, Miguel Angel Merlos Rodrigo

**Affiliations:** 1 Department of Chemistry and Biochemistry, Mendel University in Brno, Brno, Czech Republic; 2 Therapeutic Innovation Research Center (NUPIT), Federal University of Pernambuco, Recife-PE, Brazil

**Keywords:** Biochemistry, Cell biology

## Abstract

Metallothioneins (MTs) are small cysteine-rich intracellular proteins. The best-known biological functions of MTs are sequestration of metal ions and maintenance of redox homeostasis. Despite these protective functions, it has been demonstrated that MTs are involved in tumorigenesis, cellular differentiation, drug resistance, and metabolic disorders such as diabetes and obesity, in which MTs expression is substantially deregulated in adipose tissue. In addition, many studies have experimentally evidenced a possible role of MTs in the development of diabetes. Given the rich biochemical properties of MTs, it can be concluded that they are involved in several aspects of development and progression of obesity and diabetes. Thus, evaluation of expression of MTs could serve as biomarker to personalize available therapeutic interventions and possibly to develop novel advanced therapeutic modalities. Overall, the purpose of this review is analyze and review the latest studies aimed on the multiple roles of MTs in metabolic disorders, possible use of MTs as obesity and diabetes biomarkers and the role of MTs in cardioprotection during diabetes progression.

## Introduction

Diseases linked to obesity, such as type 2 diabetes mellitus (T2DM), cancer, and cardiovascular diseases, are caused by excessive energy and fat consumption [1–3]. Obesity is defined by an increase in the amount of adipose tissue typically accompanied by the generation and/or accumulation of additional adipocytes [4]. An essential element in the progression of cardiovascular disorders is the effect of obesity on adipokines, which can result to an increase in adipokines and a decrease in anti-adipokines. These changes in adipokines regulation contribute to the development of cardiovascular disease [5]. The progression of obesity involves three major processes: (*i*) formation of new adipocytes from preadipocyte precursor cells, (*ii*) maturation of adipocytes, and (*iii*) cell enlargement due to the accumulation of excess lipids in adipocytes [6]. The common link between diabetes mellitus and obesity-related issues is insulin resistance, which affects fat deposition in adipose tissue [7].

T2DM develops due to insulin’s inhibitory effect on liver glucose production or due to different defects in insulin secretion. Brief exposure to free fatty acids (FFAs) can enhance insulin secretion in pancreatic islets [8]. However, prolonged exposure to FFAs hampers glucose-induced insulin secretion and insulin production, potentially resulting in apoptosis-mediated β-cell death. Moreover, elevated levels of non-esterified fatty acids contribute to β-cell dysfunction and loss and the incidence of toxicity due to the formation of H_2_O_2_ in peroxisomes [9]. Patients with diabetes often develop cardiomyopathy, which makes them more vulnerable to heart failure and increases their risk of death from myocardial infarction, regardless of whether they have a vascular disease. Preclinical animal research has suggested that the progression of diabetic cardiomyopathy (DCM) is affected by the presence of reactive oxygen species (ROS). It is assumed that the oxidative stress coming from hydrogen peroxide occurrence can be associated with the number of antioxidative mechanisms including those based on -SH containing molecules. In 1957, Margoshes and Vallee discovered low-molecular-mass sulfhydryl-rich proteins, the metallothioneins (MTs) [10]. The molecular weights of MTs range from 6 to 7 kDa, and they are characterized by the abundancy of thiol groups, with ~30% of the amino acid being cysteine [11]. It is noteworthy that, formerly, MTs were considered to be proteins involved in the detoxification of non-essential and excess essential metals, homeostasis, and the scavenging of free radicals [12–15].

In the last decade, it has been demonstrated that MTs are induced in adipose tissue during the development of obesity [16]. In addition, several studies have evidenced the role of adipose tissue MTs in the development of obesity and T2DM [17–20]. These studies showed that the translation of zinc (Zn)-induced or genetically upregulated MTs in the pancreas prevented the development of spontaneous or chemically-induced diabetes [21]. MTs have also been shown to exhibit the potential to prevent obesity-related diseases through the scavenging of superoxide and endoplasmic reticulum stress [6]. Importantly, it has been shown that fasting/refeeding was able to markedly affect the gene expression of *MTs* in rat livers [22]. Of note is that MTs have been effective in reversing many aspects of DCM, and the overexpression of MTs has efficiently reduced the degree of cardiac damage [23].

**Fig. 1:**
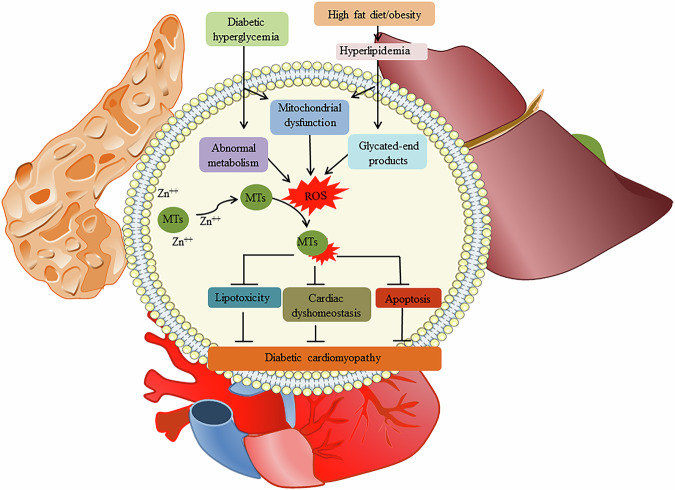
The scavenging of ROS by MTs is beneficial to the prevention of diabetic hyperglycemia, obesity, and the defense against cardiomyopathy. ROS participate in lipotoxicity, extracellular matrix buildup, disrupted calcium homeostasis, and programmed cell death.

Furthermore, evidence indicates that upregulating MTs successfully prevents oxidized glutathione (GSSG) fluctuations and mitigates multiple factors of DCM. Many agents that stimulate ROS generation have been shown to be counteracted by MTs. The scavenging of ROS by MTs is beneficial to the prevention of diabetic hyperglycemia, obesity, and the defense against cardiomyopathy, as depicted in Fig. 1.

Over the past decade, wealth of reviews and studies have emerged emphasizing the significance of MTs in various diseases, including cancer. However, only a limited number of studies have dedicated attention to investigating the precise role of MTs in obesity and diabetes [24, 25] (for a summary, see Table 1). Therefore, in our exploration of this subject, we aim to shed light on both the current advancements and future prospects that lead to innovative precision medicine approaches. Specifically, we focused on the role of MTs in people with diabetes and obesity who are also affected by COVID-19, uncovering new insights and potential avenues for therapeutic interventions.

**Table 1:** Summary of studies focused on the specific role of MTs in obesity and diabetes.

Disease/State	Function	MT Isoform	Type of study/Methodology	Year	Reference
Cardiac insulin resistance	MT prevents of DCM	MT	*Male MT cardiac-specific transgenic (MT-TG) mice/cardiac oxidative stress and fibrosis assay	2020	[20]
Diabetes	MT protects against high glucose-induced ROS	MT-1 MT-2	*Knockout (MT^−/−^) mice/light microscopy/immunofluorescence/ELISA	2014	[128]
	Elevated levels of MT in insulin-deficient rats with diabetes	MT	*Streptozotocin-induced rats	1988	[123]
	MT has defense effects against streptozotocin-induced diabetes in rats.	MT	*Lipid peroxidation/western blot	1994	[125]
	MTs protect the kidney from oxidative stress against diabetic nephropathy	MT-1 MT-2	*Immunofluorescence/protein expression in mProx cells	2011	[130]
	MT-1 negatively regulates glucose-stimulated insulin secretion	MT-1	*MT-1–MT-2 double-knockout (KO) mice/transgenic mice overexpressing MT-1/human islets	2019	[129]
	MT-mediated hepatic protection of Zn against T2DM	MT	*MT-KO mice/western blot	2021	[154]
Diabetic cardiomyopathy	MT prevention of diabetes-induced pathological changes in cardiac tissues	MT	*MT-TG mice/western blotting/mass spectrometry	2013	[17]
	Overexpression of MT reduces DCM	MT	*OVE26 mice with diabetes/RNA analysis	2002	[148]
	MT protects the heart from diabetes-induced injury.	MT-2	*MT-TG mice/northern blot assay/western blot	2005	[143]
	MT prevents of DCM	MT-1	*Male C57BL/6J mice/serum triglyceride/northern blotting	2006	[141]
	MT prevents the development of cardiomyopathy	MT	*Cardiac-specific MT-overexpressing transgenic (MT-TG) and catalase-overexpressing transgenic (CAT-TG) mice	2008	[151]
	Higher MT levels in presence of cardiovascular disease complications in people with diabetes.	MT-1A	## Study population/MT-1A polymorphism analysis	2008	[144]
	Association of +1245 MT-1A polymorphisms with T2DM in Indian population	MT-1A	## Study population/MT-1A polymorphism analysis	2021	[145]
	MT prevents diabetic activation of GSK-3β-mediated pathogenesis	MT	*MT-TG mice/qRT-PCR/western blotting	2009	[155]
	MT prevents both diabetes- and Ang II-induced cardiac ER stress	MT	*MT-TG/western blotting	2009	[150]
	MT induces cardiac protection in heart of people with diabetes	MT	*Cardiac-specific MT-overexpressing transgenic (MT-TG) mice/qRT-PCR/ELISA	2012	[152]
	The cardioprotective effects of Zn are MT-dependent.	MT	*Male MT-KO mice/western blotting	2014	[149]
	MT prevents intermittent hypoxia-induced ER stress in the heart	MT	*Male MT-TG mice/TUNEL/western blot	2014	[134]
	Cardiac MT overexpression rescues DCM in Akt2-knockout mice	MT	*Male and female MT-TG/Akt2-KO mice/Western blotting	2021	[142]
	MT prevents cardiac pathological changes in diabetes by modulating nitration and inactivation of cardiac ATP synthase	MT-1 MT-2	*MT-TG mice/western blot analysis/HPLC	2014	[133]
	Nrf2 and MT prevent DCM	MT	*MT-KO mice/echocardiography/western blot analysis/qRT-PCR	2017	[19]
Fasting stress	Leptin induces MT during fasting	MT	*MT-null (129 Sv MT^−/−^) mice/measurement of MT content	2002	[89]
Healthy	The ability of MT-null mice to develop obesity	MT-1 MT-2	*MT-null mice/microarray/northern blot	2005	[66]
High-fat diet–cardiomyopathy	MT prevents high-fat diet-induced cardiac contractile dysfunction	MT	*Male FVB and cardiac-specific MT overexpression transgenic mice	2007	[153]
Diabetic retinopathy	MT is overexpressed in Akimba mouse, a model of diabetic retinopathy	MT	*Male Akimba mice retina/single-cell RNA sequencing/immunohistochemistry	2020	[156]
Obesity	MT prevents against HFD-induced obesity	MT	*Female MT-1- and MT-2-null/body weight, fat mass, and plasma cholesterol analysis	2010	[126]
	Expression of the Zn transporter genes and MT in women with obesity	MT-1 MT-2	## Women with obesity/qRT-PCR	2006	[69]
	Cd modulates adipocyte functions in MT-null mice	MT	*MT-null mice/immunoblot analysis/RT-PCR analysis	2013	[83]
	MTs protect against high-fat diet-induced consequences in MT knockout mice	MT	*Metabolomics	2015	[4]
	Zn rescues obesity-induced cardiac hypertrophy *via* stimulating MT	MT	*C57BL/6J male mice/western blotting/qRT-PCR	2017	[80]
	Increased expression and methylation of *MT* genes in myoblasts from people who are not overweight	MT-1A, -1E, -1F, -1G, -1H, -1M, -1X MT-2A	# Primary human myoblasts/DNA methylation and mRNA expression arrays	2017	[54]
	Subpopulation of potential dysfunctional CD8^+^ T cells with cell-specific expression of *MT* genes	MT-1F MT-1G MT-2A	# Adipose tissue of people with obesity/single-cell RNA sequencing	2020	[196]
	Association with Zn nutritional status and MT in adults	MT	## Flow cytometric determination of MT levels	2010	[161]
Obesity – White adipose tissue (WAT)	*MT* gene expression in WAT	MT-2A	# The level of MT-2A mRNA	2002	[18]
	*MT-3* as a highly hypoxia-inducible gene in human adipocytes	MT-3	# Human adipocytes/PCR	2008	[56]
	Cd reduces adipocyte size and expression levels of adiponectin and Peg1/Mest in adipose tissue	MT-1	*Male Slc:ICR mice/histopathological examination/semiquantitative RT-PCR	2010	[76]
Obesity diabetes	Fasting/refeeding may influence expression of *MT* genes in WAT of rats	MT-1 MT-2 MT-3	*Male Wistar rats/real-time PCR/western blots/serum insulin concentration assay	2016	[22]
	The activation of the insulin signaling pathway	MT-1 MT-2	*WT and MT-KO mice/small interfering RNA/immunocytochemistry	2017	[16]
Type 1 diabetes	Zn treatment prevents type 1 diabetes-induced hepatic oxidative damage	MT	*OVE26 mice/immunohistochemical staining/western blotting	2015	[81]
	MT protein expression increases in rats with diabetes	MT	*Male Wistar rats/DAB immunostaining/western blotting	2018	[122]
Type 2 diabetes	MT proteins as markers and potential targets in T2DM	MT-2A	## T2DM and people without diabetes with overweight/qRT-PCR	2013	[127]
Acute cold exposure	Acute cold increased of MT-1 mRNA in brown adipose tissue	MT-1	*Male Wistar rats/northern blotting	2002	[197]
MASLD	Increased expression of MT-1 and MT-2 after ethanol and/or high-fat diet in a rat model of MASLD	MT-1 MT-2	*Male Wistar rats consuming ethanol and/or high-fat diet/RT-qPCR	2014	[47]
	Nonderepressible 2 regulates MTs expression	MT-1 MT-2	*GCN2^−/−^ and C57BL/6j mice consuming high-fat diet/western blotting/ELISA	2018	[46]
Menopause	Upregulation of MT-1E in visceral adipose tissue of postmenopausal women	MT-1E	## Visceral and subcutaneous adipose tissue/microarrays/qRT-PCR	2011	[198]

Type of study: *preclinical (mouse/rat), # clinical (human), and ## human (epidemiology).

## MT isoforms and regulation

The majority of MTs found in animals are compact proteins that are rich in cysteine and are capable of attaching to metals. They are characterized by their limited molecular weights and contain a single peptide chain consisting of 61–68 amino acids. Of these amino acids, 20 are cysteines that are distributed in two domains, known as α- and β-clusters, which have the ability to bind up to 7 divalent metal ions [26–28]. The binding of metals to MTs is achieved by through interactions with the thiol group present in the Cys residues. In contrast, MTs without any bound metal (known as apo-MTs or thioneins) possess a mainly disordered molecular structure, which renders them extremely susceptible to proteolytic degradation [29].

From the point of view of the MTs expression, the functional *MT* genes in humans are located on chromosome 16q13 [30]. There are more than 10 functional (sub)isoforms of MTs, which are divided into four isoform classes, designated MT-1 to MT-4. MT-1 and MT-2 are the two isoforms prevalent in the majority of cells and tissues. Microheterogeneity of MT-1 has been emphasized by the identification of seven functional genes, A, B, C, D, E, F, G, H, and X, which all encode proteins involved in metal binding. The rest of the MTs are encoded with single gene [31]. The presence of two significant elements in the regulatory region of the *MT-1* and *MT-2* genes, namely, the TATA box (serving as the core promoter element) and multiple cis-acting response elements, including metal-responsive elements (MREs) and antioxidant-responsive elements (AREs), drives their expression [32]. MTs promoter is activated by a range of ions *via* metal-responsive elements (MREs), but only Zn is directed towards the activation and binding of the metal-regulatory protein, MRE-binding transcription factor-1 (MTF-1). The DNA-binding activity of MTF-1 can be reversibly activated in response to fluctuations in free intracellular Zn^++^ concentrations [33]. MTF-1, a transcription factor with six Zn fingers, is a central player in the induction of MT-1 transcription in response to metals and oxidative stress. The MRE located in the region near the MT promoter is targeted by MTF-1, whose expression is also inducible. It is noteworthy that Zn is the sole metal known to stimulate MTF-1, although it has been reported that MTF-1 can be activated by free radicals [34]. By contrast, canonical Cys2His2 Zn fingers typically bind to Zn^++^ with a higher affinity (10^−12^–10^−9 ^M). The affinities of all six Zn fingers for Zn in MTF-1 are similar, varying by only 10- to 50-fold [35, 36].

Based on these facts, it can be concluded that MTF-1 plays a crucial role in maintaining Zn^++^ homeostasis through its involvement in the Zn^++^-responsive transcription of *ZnT1* and *ZnT2*, while simultaneously suppressing the expression of Zip10 [37, 38]. The genomes of mammals encode 14 Zn transporter proteins (*ZIPs*) and 9 *ZnT* transporter genes, each of which exhibits distinct cellular and subcellular localizations as well as unique tissue-specific, developmental, and stimulus-responsive expression patterns.

**Fig. 2:**
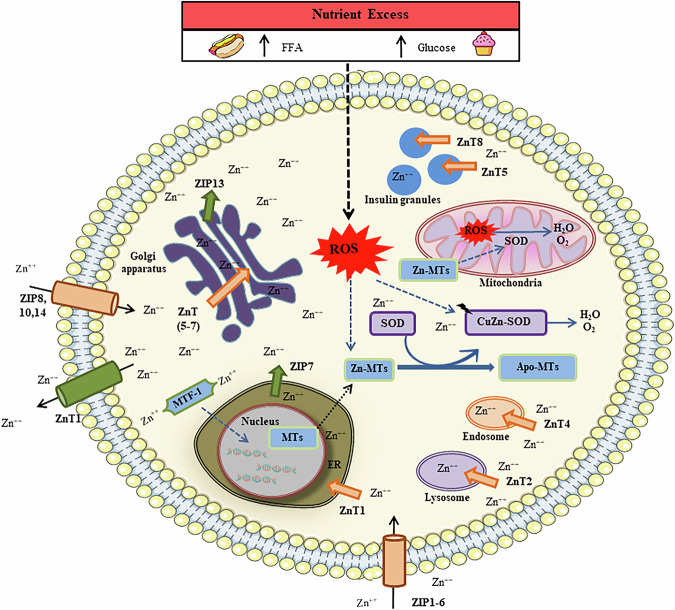
Cellular localization of Zn^++^ transporters and MTs. The arrows indicate the predicted direction of Zn^++^ mobilization, where ER refers to the endoplasmic reticulum.

Furthermore, the activation of Zn^++^-MT diminishes the harmful effects of excessive nutrient intake-induced oxidative stress generated by free radical [39, 40]. The connections across Zn^++^, MTs, and nutrient excess are highlighted in Fig. 2.

Interestingly, changes in Zn metabolism have been observed in both animals with and without diabetes. There is evidence that Zn supplementation is beneficial in people and animals with diabetes, suggesting that this metal may have a potential role in treating diabetes in the future. This claim is supported by the identification of ZnT8, a protein responsible for Zn regulation that may be involved in the process leading to T1DM and T2DM [41, 42]. Additionally, a common *ZnT8* gene (*SLC30A8*) polymorphism increases the risk of T2DM, and rare mutations may present protective effects. In T1DM, autoantibodies show specificity for binding two variants of ZnT8 (R or W at amino acid 325) dictated by a polymorphism in *SLC30A8* [43].

### MTs in high-fat diet and obesity

Based on epidemiological evidence, it can be concluded that ingestion of a diet high in fat is linked to the development of obesity. Furthermore, it has been observed that the greater the dietary fat consumed, the greater the likelihood of developing obesity [44]. Thus, we further discuss and describe the potential role of MTs in the clinical management of obesity-related diseases.

### MTs in high-fat diet

#### MTs in lipid metabolism

Obesity and metabolic disorders are frequent consequences of high-fat diet feeding. Approximately 15–30% of the population worldwide is affected by metabolic dysfunction-associated steatotic liver disease (MASLD), formerly known as nonalcoholic fatty liver disease (NAFLD) [45]. MASLD is the most common chronic liver disease and comprises histopathological disorders that range from the accumulation of triglycerides in the liver (steatosis) to non-alcoholic steatohepatitis (MASH) characterized by the presence of inflammation and fibrosis in the liver [14]. Liver steatosis results from an excessive delivery of FFAs from adipose tissue into the liver accompanied by insufficient lipid degradation as well as from an imbalance between the new lipid synthesis and catabolism. Catabolism of lipids is thus highly dependent on mitochondrial metabolism in the liver. An increased mitochondrial respiratory activity in the liver therefore speeds up the degradation and prevents the accumulation of fatty acids in the liver, which prevents the development of fibrosis in MASH [15]. The impact of general control nonderepressible 2 (GCN2) kinase, a regulator-feeding behavior, on the development of MASLD was further investigated by Liu et al. [46]. GCN2 is involved in maintaining the homeostasis of amino acids and regulating the hepatic lipid metabolism in a diet-dependent manner. In GCN2-deficient mice fed a high-fat diet, a downregulation of MT-1 and MT-2 was found together with obesity and hepatic steatosis.

#### Oxidative stress and zinc/copper homeostasis in MASLD

The development of human MASLD is thought to be closely linked to increased oxidative stress, with Cu/Zn-SOD being one of the enzymes that counteract this stress. This enzyme requires sufficient copper availability, indicating a correlation between copper availability and antioxidant defenses in MASLD [11]. In a rat model of MASLD, an increased expression of MT-1 and MT-2 mRNA was found as a consequence of binge alcohol consumption and high-fat diets [47]. Our understanding of the roles of MTs and MASLD in the maintenance of Zn homeostasis in the liver has been slowly evolving. The transport of Cu/Zn in hepatocytes can be divided into three steps: (i) uptake, (ii) intracellular distribution, and (iii) utilization and export, which all may be facilitated by MTs. Patients with disorders associated with the increased copper concentration in liver have significantly elevated concentrations of MTs in their plasma [13].

#### MTs in late-stage MASLD and hepatocellular carcinoma

Furthermore, it has been shown that MASH can lead to a significant percentage of cirrhosis cases that cannot be attributed to a specific cause (idiopathic or cryptogenic). Late-stage MASLD can also result in hepatocellular carcinoma (HCC), which is a serious health condition.

HCC is the most prevalent form of primary liver cancer and ranks as the second leading cause of cancer-related death globally [48]. The presence of diabetes and obesity has been firmly established as separate factors that increase the risk of developing HCC. In HCC, the downregulation of MT expression might play a role in carcinogenesis [49], but the biological and clinical significance of this downregulation is still uncertain. However, many studies have shown that upregulation of MT are linked to the acquisition of chemoresistance in HCC to various anticancer drugs [50].

### MTs in obesity

#### MTs in obesity and public health impact

According to the World Health Organization, obesity has become a significant public health threat of the 21^st^ century and its prevalence has tripled in developed countries since the 1980s, making it one of the greatest health challenges we face today, and the number of those affected continues to rise at an alarming rate. The Centers for Disease Control and Prevention estimated that during 2017–2018, 42.4% (99.4 million) of American adults and 18.5% (13.7 million) of American children and adolescents were clinically people with overweight. Along with physical disabilities and psychological issues, there is a considerable increase in the risk of diabetes, cancer, and cardiovascular diseases associated with obesity [51]. Deaths from cardiovascular diseases are sometimes involved in obesity, which is also associated with the development of diabetes and atherosclerosis-related diseases. The development of these diseases increases the mortality rate in cardiac patients [52].

#### Mechanisms of DNA damage, inflammation, and hypoxia in obesity

Dysregulation of DNA repair pathways and DNA damage induced by obesity can result in an increased mutation rate and may cause healthy tissues to transform into cancerous tissues. The DNA damage in the lymphocytes of people with obesity was found to be about twice as high as in the lymphocytes of normal-weight subjects; thus, studies have reported a correlation between DNA damage and body mass index (BMI) [53]. Changes in the patterns of DNA methylation and MT expression across the genome were detected during the differentiation of human myoblasts from people with obesity, according to the study [54]. Genetic factors are believed to play a role in determining the susceptibility to obesity, i.e., due to gene mutations of melanocortin-4, proopiomelanocortin, and leptin receptors etc. or a protein deficiency in interleukins, angiopoietin and others [55]. An inflammatory response and angiogenesis are developed during obesity as well. There is a direct link between hypoxia in adipose tissue and obesity. Moreover, hypoxia leads to increased glucose uptake by human adipocytes. Wang et al. [56] showed that the *MT-3* gene protects against hypoxic stress in adipocytes. This suggests that MT-3’s main function in fat tissue is the same as that of astrocytes in the brain, which protect this tissue from hypoxic damage.

#### MTs and oxidative stress in obesity

Many studies have shown that chronic energy overload in obesity results in enhanced ROS production and inflammation [24, 25]. According to the available data, the sources of ROS may vary based on the stage of obesity. In the initial stages of obesity, the uptake of fatty acids and glucose by adipocytes leads to the activation of NOX4, the major isoform of NADPH oxidase found in adipocytes, resulting in the increased production of ROS [57–59]. Furthermore, chronic inflammation associated with obesity plays a significant role in the development of DNA lesions [60]. Disturbances in glucose homeostasis and mitochondrial dysfunction leading to FFA oxidation failure are also related to obesity [59, 61, 62].

Recently, a study highlighted that overexpressing MT-3 reduces ROS levels in early adipocyte differentiation, while antimycin A treatment restores ROS levels. MT-3 knockdown increases ROS levels, lowered by the antioxidant N-acetylcysteine. These findings reveal MT-3’s previously unknown role in 3T3-L1 cell differentiation, suggesting it as a potential target for obesity treatment. MT-3 acts as an inhibitor of adipocyte differentiation by suppressing adipogenic transcription factors, including C/EBP family members and PPARγ, and downregulating PPARγ‘s transcriptional activity, with its regulatory impact linked to ROS scavenging [63]. Obesity induces an endoplasmic reticulum (ER) stress, leading to the development of diabetes by causing insulin resistance [64]. Moreover, a Zn deficiency upregulates the mammalian ER stress, which indicates the induction of MT synthesis and enhanced stress response [65]. MTs could play a crucial role in metabolism and the utilization of fat deposits because, in mitochondria, MTs have an antioxidant role against ROS, thus increasing the metabolism and modulating cellular respiration [66]. MTs could also attenuate the stress in this organelle by providing intramolecular Zn to recover the normal folding of ER proteins. It is assumed that, through modulation of ER stress, the Zn-MT complex is involved in regulating the development of insulin and leptin resistance and obesity.

#### MTs and metal homeostasis in obesity

Obesity is also strongly influenced by intracellular Zn homeostasis. Several studies assessed the biochemical parameters related to people with overweight and observed that they had an alteration of nutritional status in relation to Zn, with lower concentrations in their plasma, erythrocytes, and serum [67]. Furthermore, low Zn concentrations are associated with alterations in the hormone metabolism (insulin and thyroid hormones) of the adipose tissue in people with obesity [68–70]. Numerous studies have examined the link between Zn transporters and adipose tissue metabolism [71]. Recently, a study by Fukunaka et al. has clarified the role of the Zn transporter ZIP13 in regulating the biogenesis of beige adipocytes, highlighting the regulation of Zn homeostasis as a potential therapeutic goal for the treatment of metabolic syndrome and obesity [72]. According to a study by Szrok et al. [22], increased levels of MT-2 and MT-1 may cause a decline in the levels of intracellular Zn finger proteins, resulting in the suppression of adipogenesis and lipid accumulation in adipose tissue. Furthermore, although MTs may not play a crucial role in preadipocyte differentiation and maturation, they have the potential to prevent high-fat diet-induced obesity by controlling the increase in adipocyte size [73]. In addition, Lindeque et al. [4] showed that *MT* knockout in mice resulted in higher body weight during the early stages of life, with a greater potential for obesity when consuming a high-fat diet. MT-encoding genes are expressed in several tissues and organs, including both brown and white adipose tissues (WAT). In addition, reducing the expression of MT increased the activation of the insulin signaling pathway, leading to elevated lipid accumulation in 3T3-L1 adipocytes [16]. In mammals, WAT is the main site for the storage of triacylglycerol. This tissue has several functions. WAT works primarily as an endocrine organ that secretes hormones such as cytokines, leptin, and secretory factors (TNF-α, IL-6, angiotensinogen, plasminogen activator inhibitor-1, IGF-1,adipsin and adiponectin). Some studies have also identified MTs and resistin-like secretory molecules as products expressed by white adipocytes [18, 74]. As mentioned above, MTs exhibit a protective role against the toxicities of metal ions [75]. Repeated exposure to cadmium (Cd) causes a reduction in the expression levels of adiponectin and resistin and also decreases the size of adipocytes in WAT [76]. In lean healthy individuals, adiponectin and resistin were normally secreted from WAT to regulate homeostasis in insulin sensitivity. A study confirmed that the expression levels of MT genes were higher in people with overweight than those of Zn transporters (ZnT-1, Zip-1, and Zip-3) [35]. Beginheick et al. [77] demonstrated an increase in cortisol and pro-inflammatory cytokines (which are able to induce MT expression) in the adipose tissue of people with obesity. This high expression induced a reduction in the Zn serum concentrations and their redistribution among different tissues [78]. For this reason, the compartmentalization and metabolism of Zn in patients with obesity have been investigated, resulting in the identification of high Zn concentrations in the liver, adipose tissue, and muscle [18]. According to these studies, people with overweight have a high concentration of glucocorticoids, which can cause high levels of MTs bound to Zn by a “sequestration process” in some specific tissues [79]. Children with overweight have a high risk of developing in adulthood with obesity, as well as other illnesses such as diabetes and cardiovascular disease. Wang et al. [80] published a study about cardiac abnormalities (cardiac hypertrophy and dysfunction) in childhood with obesity using a model of high-fat diet–induced obesity young mice. They demonstrated that cardiac pathogenic effects were exacerbated by a Zn deficiency and alleviated by its supplementation, which suggests that Zn plays a critical role in stimulating MTs to suppress the oxidative stress-activated BCL10/CARD9/p38 MAPK pathway [80]. Furthermore, recent studies have demonstrated that Zn supplementation provides some benefits to people with obesity (with and without diabetes) by enhancing insulin sensitivity and reducing inflammation and oxidative stress (by increasing MT expression), which leads to the attenuation of the destructive effect of obesity on the liver, heart, and kidneys [81].

In addition, ROS decrease the expression levels of leptin, adiponectin, and resistin [82]. Kawakami et al. [83] showed that MT plays a protective role against Cd toxicity, which is caused by free Cd ions (because most Cd in tissues is present in an MT-bound form). Moreover, the study reported an increase in the size of adipocytes in WAT and a lower expression level of resistin and adiponectin detected in MT-null mice compared to wild-type mice. This suggests that Cd has a direct effect on the acceleration of lipolysis, increasing the degradation of stored lipids and suppressing adipogenic gene expression [83]. Cd concentrations in human subcutaneous and visceral adipose tissues exhibited a negative correlation with a person’s BMI. In in vitro studies, exposing mature human adipocytes to Cd concentrations led to the altered expression of genes involved in trace element homeostasis and heavy metal detoxification. Indeed, these included the overexpression of MTs [84]. Importantly, lifelong exposure to low-dose Cd alters diet-induced MASLD [85]. There were also reported differences between the sexes in cardiac pathogenesis after lifelong Cd exposure in combination with a high-fat diet; studies showed that women were primarily negatively affected [86, 87]. It was previously shown that MTs play a protective role against the effects of caloric restriction diets and the inhibition of insulin [88]. After the initiation of a low-caloric diet in studies with mice, a reduction of leptin levels and an elevation of corticosterone (due to the anxiety responses following the withdrawal or reduction of food) were observed. Moreover, anxiety triggered an elevation in the binding activity of MT and glucocorticoid response, leading to the subsequent induction of MT synthesis [89]. MT mechanisms in response to caloric restriction and insulin inhibition are connected with aging. Supplementing Zn later in life has been found to promote healthy aging and enhance survival and extend the maximum lifespan of mice [90]. The levels of MTs bound to Zn are important to the mammalian aging process. The sequestering of Zn ions by MT will reduce the Zn bioavailability needed for an effective immune response to infectious agents [91]. Furthermore, an increased risk of dementia or neurodegenerative diseases was also associated with people with overweight and diabetes because, in these people, the amount of Zn available for crucial intracellular processes is prone to decrease [92]. *ZnT* and *ZIP* genes are also involved in intracellular Zn homeostasis in the human frontal cortex. These genes are responsible for regulating both the uptake of Zn from the extracellular environment and release of Zn from intracellular stores [90]. Multiple neuropsychological studies have suggested that Zn deficiency can potentially affect an individual’s perception, attention, and psychomotor skills. In a study conducted by Takeda et al. [93], Zn supplementation decreased the symptoms of depression in overweight depressed individuals.

#### MTs and heat shock proteins in obesity

Heat shock proteins (HSPs), a family of proteins that maintain cellular proteostasis and provide protection under stress conditions, represent a promising class of potential biochemical markers for obesity and T2DM [94–98]. Nowadays, some studies have suggested a potential co-regulation between HSPs and MTs in human diseases. Notably, one study found that several hepatic genes, including MT1 and various HSPs (Hspca, Hspcb, Hspa8), were expressed at approximately twice the normal levels in a HFD mouse model [99]. Additionally, another study reported elevated protein levels of HSP60, HSP27, HSP90, HSC70, and GRP94 in people with obesity compared to lean controls [100]. Tiss et al. demonstrated that people with obesity leads to dysregulated expression of multiple HSP components in people without diabetes; however, a three-month program of moderate physical exercise was sufficient to restore normal HSP expression in adipose tissue and concurrently reduce the inflammatory response [101]. Furthermore, the effect of exercise on hepatic gene expression in a mouse with obesity model had also been analyzed using cDNA microarrays, revealing notable changes in genes related to defense and stress responses, particularly MT1, MT2, and various HSPs [102].

### MTs in diabetes

#### Diabetes overview and global impact

Diabetes is a complex disease with multiple factors contributing to its development, significantly affecting healthcare systems as well as the quality of life and life expectancy of individuals affected by this disease [103]. Diabetes is characterized by hyperglycemia, oxidative stress and abnormalities in lipid metabolism. According to estimates, the global incidence of diabetes across all age groups was 2.8% in 2000 and is expected to rise to approximately ~4.4% by 2030. The total number of individuals with diabetes is projected to increase from 171 million in 2000 to 366 million by 2030 [104]. T2DM is predominantly found in adults; however, there has been a gradual increase in the incidence of this disease among children and adolescents in recent times [105, 106]. T2DM is characterized by insulin resistance and partial β-cell damage, while type I diabetes (T1DM) is distinguished by severe damage to pancreatic cells, leading to insulin dependence [107, 108].

#### MTs, oxidative stress and ROS in diabetes

In a recent study, it was proposed that MT-1E could be a promising target for drug development in T2DM. The study found that the enforced expression of MT-1E p.C36C stabilized glucose metabolism and led to a reduction in body weight, whereas the expression of MT-1E p.C36Y had the opposite effect [109]. It is known that both T1DM and T2DM are characterized by increased ROS production, which correlates strongly with the development of diabetes and its associated complications [110, 111]. Hyperglycemia-induced free radicals, such as ROS and hydroxyl free radicals, are responsible for the destruction of pancreatic β-cells caused by oxidative stress [112]. Exposing murine pancreatic β-cells to pro-inflammatory cytokines IL-6 and IL-1β led to changes in the cell metallome, especially a decrease in Zn and Ca content and an increase in Fe content, together with changes in the cellular distribution of these metals. In previous studies, Fe has been linked to the development of T2DM *via* the formation of ROS in the Fenton reaction [113]. Studies on diabetes treatment and prevention have mainly focused on investigating the oxidative stress mechanisms linked to diabetes and examining the effectiveness of antioxidants in averting these mechanisms. MTs may have the potential to prevent diabetes owing to their localization in metabolic organs, including the cytoplasm of pancreatic endocrine cells. Furthermore, various studies have demonstrated that *MT*-deficient mice are more vulnerable to oxidative stress-induced cellular and tissue damage as well as non-communicable diseases associated with oxidative stress [6, 114]. In summary, MTs could have a significant impact on diabetes prevention by mitigating hyperglycemia-induced oxidative stress as they safeguard pancreatic β-cells against damage from free radicals and maintain regular insulin secretion [115, 116].

#### MTs and zinc in diabetes pathophysiology

The roles of MTs in the pathophysiology of pancreatic β-cells have been summarized comprehensively by Bensellam et al. [117]. *MT* polymorphisms were used as risk indicators for a huge spectrum of diseases. For example, MT-1A and MT-2A and the misbalance of metal levels were associated with metabolic syndrome pathologies such as diabetes, high blood pressure, dyslipoproteinemia, atherosclerosis, etc. [118]. Furthermore, Zn has garnered significant attention in diabetes research [119]. Clinical and epidemiological studies have indicated a correlation between low Zn levels and diabetes [120, 121]. Additional support for this association comes from studies by Faure et al. [122] and Batista et al. [123], which revealed decreased Zn levels in the majority of patients with diabetes. Other numerous studies in various animal models have shown that Zn is effective in ameliorating T2DM-related complications such as hyperglycemia, insulin resistance, obesity, hyperlipidemia, and hypertension. MTs and Zn are implicated in pancreatic functions, but the highest levels of both occur in exocrine tissue as a consequence of strict Zn homeostasis [124]. Studies have indicated that Zn-induced overexpression of MT in pancreatic β-cells can confer notable protection against streptozotocin-induced diabetes in both rats and mice [125]. In addition, hypothalamic ER stress induced by chronic over-nutrition induces hypothalamic insulin/leptin resistance, which causes obesity and T2DM in rats [126]. Cai’s [21] study showed that MTs act as potent antioxidant adaptive proteins and Zn homeostasis regulators; thus, MTs are involved in vital functions that can help prevent the onset of diabetes, as well as complications and their associated toxic effects.

At constitutive levels, MTs might maintain the correct vascular myogenic tone, and it was also observed that MT-2 together with endothelin-1 contributed to endothelial cell protection against oxidative stress associated with high glucose concentrations [124].

#### MTs in obesity, insulin resistance, and diabetic complications

As we explained in the previous section, the link between obesity and diabetes was found in an insulin resistance factor called resistin, which inhibits adipocyte differentiation. Although the role of adipocyte-derived MTs is not fully understood, it is speculated that these stress-response and metal-binding proteins may act as antioxidants, shielding fatty acids from oxidative harm in adipocytes and during their transit from the micro-vasculature [18]. Furthermore, Haynes et al. [127] studied *MT-2A* expression in visceral adipose and subcutaneous tissues of people with obesity with and without diabetes, and they showed that *MT-2A* might be involved in the modulation of insulin resistance in fat cells; the polymorphism in this gene has been associated with hyperglycemia, increased HbA1c, and increased Zn deficiency in T2DM subjects. These results suggest that MT overexpression could have transient benefits but long-term metabolic consequences in metabolic disorders involved in insulin resistance [127]. Other studies suggested that MTs could play a crucial role in safeguarding the kidneys against ROS triggered by high glucose levels and in preventing inflammation in diabetic nephropathy [128]. Bensellam et al. [129] showed that MT-1 negatively regulates insulin secretion. The upregulation of MTs in T2DM may contribute to β-cell failure; therefore, the authors of the study suggested that the MT-1 inhibition could be a therapeutic attractive target to restore and preserve insulin secretion in patients with T2DM [129]. Furthermore, Ogawa et al. [130] suggested MT-1 and MT-2 as novel therapeutic targets against diabetic nephropathy, as the expression of these genes was increased by high glucose–induced oxidative stress in renal proximal tubular epithelial cells. In a mouse with obesity model, the activation of nuclear factor erythroid 2–related factor 2 (Nrf2) by sulforaphane led to a reduction in obesity-related glomerulopathy and an increase in MT expression in podocyte cell cultures [131]. Moreover, in insulin-deficient diabetic rats, increased levels of MT-1 and MT-2 were detected in the liver and kidneys. Furthermore, in the livers of rats with diabetes, there was an accelerated rate of MT expression and cytoplasmic MT turnover, whereas in the kidneys, only the expression rate of MTs was increased, but not their cytoplasmic turnover [123, 130]. A study by Mondragon et al. [122] revealed an increase in MT expression in the plantaris and soleus muscles of streptozotocin-induced diabetic rats. Future investigations in this area should examine the underlying pathways, such as mitochondrial alterations [122].

#### MTs and cardioprotection in diabetes

**Fig. 3:**
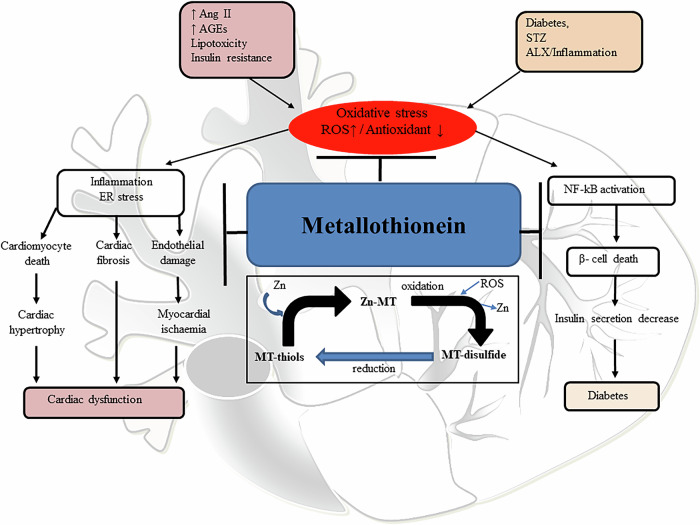
The outline of the mechanisms by which MTs prevent DCM. Ang II refers to angiotensin II, AGEs to advanced glycation end-products, ALX to receptor A lipoxin, DCM to diabetic cardiomyopathy, ER to endoplasmic reticulum, NF-κB to nuclear factor kappa-light-chain-enhancer of activated B cells, ROS to reactive oxygen species, and STZ to streptozotocin.

Several studies have demonstrated that MTs can prevent and inhibit cardiac oxidative stress, protecting against the cardiotoxicity caused by chemotherapeutic agents [132], inhibit pathological changes in the hearts of diabetes patients [133], and protect the cardiac endoplasmic reticulum from stress and apoptosis [134, 135]. Figure 3 shows the proposed mechanisms, by which MT prevents diabetes cardiomyopathy.

The aging of the population, cardiorenal metabolic syndrome, and the global obesity epidemic are the main factors that contribute to heart failure due to associated reductions in cardiac insulin metabolic signaling and insulin resistance [136–138]. To develop cardiac insulin resistance, major factors such as hyperglycemia, hyperlipidemia, oxidative stress, inappropriate activation of sympathetic nervous system and the renin-angiotensin II-aldosterone system (RAAS), and dysregulated secretion of adipokines/cytokines are all significant contributors [139]. In addition, diabetes alters the antioxidant capacity of the heart, which is shown in the decreases in enzymatic (catalase, superoxide dismutase, glutathione peroxidase etc.) and non-enzymatic antioxidant defenses (vitamins A, E, or C) [140].

Recent studies have demonstrated that MTs effectively prevent early phase cardiac cell death, which is primarily responsible for the onset of DCM [141, 142]. This was also verified by Song et al. [143], who showed that cardiac overexpression of MTs significantly protects the heart from diabetes-induced injury. Song et al. [143] also proposed that MT induction in the liver or heart due to diabetes is not associated with metal ions content. Instead, they suggested that the development of diabetic cardiovascular problems might be related to abnormal homeostasis of Zn or Cu. In the context of atherosclerosis and aging, there is a notable increase in inflammation and oxidative stress, in which high MT levels are associated with lower Zn availability. One of the oxidants capable of inducing the release of Zn from MTs is nitric oxide (NO). The study of *MT* polymorphisms may be of utmost importance, as Giacconi et al. [144] demonstrated that a mutation in *MT-1A* was associated with diabetes and its cardiovascular complications. *MT-1A* polymorphism was also associated with the risk of T2DM in the Indian population in a work by Kumar et al. [145], and *MT-1A* and *MT-2A* polymorphisms were associated with T2DM and coronary artery disease in the study of a Bulgarian cohort by Kozarova et al. [146].

In the hearts of subjects with diabetes, the evidence of oxidative stress is a significant increase in GSSG and decreased levels of glutathione, where MTs play a preventative role against DCM by maintaining the cellular redox status mainly through binding and releasing Zn. In response to NO and peroxynitrite, MTs release Zn, which induces MTF-1, and this increases the expression of protective genes by scavenging some ROS and reactive nitrogen species [147]. Moreover, Liang et al. [148] revealed that the overexpression of MTs prevents diabetes-induced changes in GSSG levels as well as lowers several markers related to DCM.

Adequate Zn supplementation and regular monitoring of the Zn levels are important to slow the development of DCM. Sun et al. [149] suggested that Akt metabolism-mediated glucose signaling through Zn necessitates MTs, highlighting the significance of MT-dependence for the cardioprotective effects of Zn. This is particularly crucial because patients with *Akt2* gene deficiency or insulin resistance may require Zn supplementation [149].

In summary, Zn supplementation was shown to be effective in inducing MT to preserve cardiac Akt2 signaling and prevent DCM. These results suggest that the upregulation of tribbles homolog 3 protein (TRB3) due to diabetes results in impaired cardiac glucose metabolism *via* the inhibition of cardiac Akt2 function. However, MT activity toward cardiac Akt2 function by inhibiting TRB3 can prevent DCM [20]. Furthermore, a study revealed that tunicamycin administration in the ER caused a significant increase in cell death exclusively in wild-type mice and that administering antioxidants prior to angiotensin II prevented ER stress and apoptosis in cultured cardiac cells. These findings indicate the existence of ER stress in the hearts of individuals with diabetes, potentially leading to cardiac cell death. MTs can prevent both diabetes- and angiotensin (AngII)-induced cardiac ER stress and associated cell death, likely due to their antioxidant properties, which may be responsible for MT-induced prevention of DCM [150]. Moreover, Zhou et al. [151] observed that in both subjects with and without diabetes, MTs play a cardioprotective role against Ang II-induced cardiac cell death and nitrosative damage. MTs suppress Ang II-induced NOX-dependent nitrosative damage and cell death faster in diabetic than non-diabetic subjects, besides preventing the late development of Ang II-induced cardiomyopathy [151]. Furthermore, Xue et al. [152] showed that MTs’ activation of HIF-1 plays a role in cardiac protection in patients with diabetes. Moreover, this study revealed for the first time that the downregulation of the mitochondrial biogenesis coactivator PGC-1α and its downstream nuclear factors are linked to mtDNA content in high-fat diet-induced obesity, muscle density loss, mitochondrial and myocardial contractile dysfunction, ROS accumulation, and intracellular Ca^2+^ signaling. These findings support the theory that high-fat diets (potentially causing insulin resistance and T2DM) hinder mitochondrial biogenesis, which could clarify the development of anomalous myocardial performance in cases of obesity, insulin resistance, and fully developed diabetes [153]. In addition, Gu et al. [19] presented the direct confirmation of Nrf2’s crucial function in protecting against T2DM-induced DCM using sulforaphane (SFN). As Nrf2 functions merely as a nuclear transcription element and cannot directly hinder oxidative damage, SFN-induced Nrf2-mediated protection from diabetes requires Nrf2 downstream genes. The clinical relevance of the correlation between Nrf2 and *MT* gene polymorphisms and various illnesses, notably cardiovascular diseases, is highly significant. Hence, investigating intervention approaches that control Nrf2 and/or MTs in patients with genetic polymorphisms, potentially through SFN and/or Zn, may be advantageous for protecting the heart. The significant prevention of the development of DCM is due to the attenuation of early-phase cardiac cell death by MTs [19]. In addition, other studies indicated that Zn supplementation prevented T2DM-induced liver injury through the activation of the Nrf2-MT-mediated antioxidative pathway [154]. This process was mediated by MT suppression of mitochondrial oxidative stress. This phenomenon was also evidenced by Wang et al. [141], who observed that cardiac-specific overexpression of MT in transgenic mice confers high resistance to cardiomyopathy induced by diabetes. The overexpression of MTs in the heart alleviated the oxidative stress and activated the transcription factor c-Jun, as well as impaired insulin signaling [141].

Cong et al. [133] revealed that the main action by which MT protects against DCM is *via* limiting superoxide production alongside its correlated nitrosative injury. Another study hypothesized that MTs might protect the heart from diabetes-linked alterations by impeding nitration of SCOT Trp374, conserving its enzymatic function in mitochondrial energy metabolism [20]. The authors of this study showed that Zn supplementation produced MT overexpression in the heart, resulting in the prevention of diabetes-triggered ATP synthase nitration, inactivation, inflammation, and profibrotic response. Furthermore, Zn supplementation was effective in reducing ethanol-induced hepatic nitration and depletion of Zn, thereby protecting the liver from alcohol-induced injury [133]. Additionally, Zn therapy decreased the likelihood of vascular conditions, such as retinopathy, nephropathy, and neuropathy, among people with diabetes. Wang et al. [155] examined the possible mediation of MT cardiac protection against diabetes by glycogen synthase kinase 3 inactivation, which is a crucial promoter of energy metabolic dysfunction and pathological restructuring in the heart that arises due to diabetes. The retinal upregulation of MTs, together with other proteins connected to metal ions and oxidative stress, was revealed in a mouse model of diabetic retinopathy, which displays the vascular pathologies and other complications of diabetic retinopathy [156].

Another study identified that after myocardial infarction in mice; MTs helped maintain cardiac function, minimized infarct size, and lowered the occurrence of cardiomyocyte apoptosis. The significance of MTs during myocardial infarction extends beyond their ability to scavenge ROS. Rather, MTs were shown to have a novel function in protecting the heart by mitigating myocardial infarction-induced cardiomyocyte apoptosis through the activation of the mTORC2/FoxO3a/Bim pathway rather than merely through their ROS sequestering ability. Therefore, an MT-modulated mTORC2/FoxO3a/Bim pathway may have a crucial role to play in the management of myocardial infarction, as a result [157]. Thus far, the knowledge of MT mechanisms against oxidative stress may achieve a reduction in the mortality of patients with ischemic heart disease, particularly in cases with diabetes mellitus as a co-morbidity.

## The pharmacological benefits of modifying MTs in obesity and diabetes

Based on all the information in the previous chapter, we briefly provide a summary of the existing evidence regarding the pharmacological benefits of modifying MTs in obesity and diabetes. The evidence for the preventive effect of MT on diabetes in in vivo models, as demonstrated under various experimental conditions, indicates that Zn pretreatment (e.g., i.p. intraperitoneal injection of 10 mg/kg [125], drinking water with 25 mM for 1 weeks [158], and dietary supplementation with 1000 ppm for 2 weeks [159]) effectively increased pancreatic MT levels and provided protective outcomes in rats and mice with STZ-induced diabetes, with results ranging from moderate to significant protection. Similar protective effects were observed in genetically enhanced transgenic mice (STZ, 1 × 200 mg/kg) for MT, which resulted in a 20-fold increase in MT expression and significant prevention of STZ-induced diabetes [160].

Moreover, Costarelli et al. carried out a clinical study in a total of 223 overweight/people with obesity (125 females and 98 males). These subjects were subdivided in two groups: Group 1 (*n* = 100) with low-Zn dietary intake (<7 mg/day for females and <9.5 for males) and Group 2 (*n* = 123) with normal-Zn dietary intake (≥7 mg/day for females and ≥9.5 for males). Interestingly, individuals with low dietary Zn intake show both reduced MTs production and a limited ability to release Zn from MTs, indicating a diminished capacity to respond to oxidative stress and inflammation [161]. This impaired response may contribute to the development of obesity or exacerbate existing obesity. Another study providing pharmacological evidence of altered MTs regulation and its impact on Zn homeostasis in obesity was conducted by Mota Martins et al. This case-control study included 80 women, divided into two groups: women with obesity group (*n* = 40) and a normal-weight control group (*n* = 40). Dietary Zn intake was assessed using a three-day food record, and compared against the Estimated Average Requirement (EAR) of 6.8 mg/day for females. The findings indicated that abdominal fat accumulation and increased visceral adiposity were associated with altered cortisol metabolism, which in turn contributed to elevated MTs expression and subsequent disruptions in Zn metabolism [162].

### Is there brief evidence of MTs related to COVID-19 in patients with diabetes?

Following the identification of initial cases in Wuhan, China, in December 2019, the severe acute respiratory syndrome coronavirus 2 has continued to spread worldwide, with COVID-19 declared a pandemic on 11 March 2020 [163–165]. The COVID-19 pandemic has so far infected millions, resulting in the death of over a million people as of March 2021 [166, 167]. There is rapidly emerging evidence that highlights obesity and T2DM as comorbidities of SARS development in COVID-19 [168, 169]. Based on the above-mentioned statistics of several diabetes patients, a significant proportion of the population may be at higher risk of adverse outcomes due to COVID-19. An increased risk for severe forms of COVID-19 was also reported in older individuals and patients with cardiovascular diseases and DCM [170, 171].

Cohort studies have shown that people with diabetes are more likely to develop severe COVID-19 and require more intensive care, including the need for a ventilator and access to an intensive care unit [172]. The exact pathophysiological mechanisms that heighten the risk of severe disease and mortality from COVID-19 in individuals with diabetes remain unclear [173–175]. Overall, individuals with diabetes face a higher risk of infections compared to the general population, with pneumonia being a leading cause of mortality among them [176, 177]. This is noteworthy when one considers the fact that COVID-19 predominantly affects the respiratory system, resulting in pneumonia and acute respiratory distress syndrome [178]. Furthermore, massive studies of almost 200,000 people found that those who suffered from COVID-19 have a greater risk of developing diabetes. Like other viruses, COVID-19 might damage pancreatic cells that produce insulin, thus triggering T1DM mainly in children and young people [179, 180]. A detailed study by Boudreault et al. [181] demonstrated that low levels of Zn in plasma predispose a person to ventilator-induced injury in intensive care because Zn is related to the role of the MT system in lung protection. As we explained at the beginning of this review, *MT* expression is induced by Zn through the binding of Zn to MTF-1, which binds to MREs in the promoter of the *MT* gene [33]. Zn is an essential micronutrient crucial for immune system function, serving as a signaling molecule. It acts as an anti-inflammatory agent, functions as an antioxidant, stabilizes cell membranes, and induces MT [182]. Various evidence suggests that MT expression increases in response to bacterial and viral infections, and it is believed that the upregulated biosynthesis of human MTs may play a significant role in nutritional immunity during active infection [183, 184]. Moreover, several studies have concluded that Zn is considered a potential supportive treatment in the therapy of COVID-19 infection due to its immune modulatory effect as well as its direct antiviral effect [185]. Su et al. [186] utilized computational methods to understand immune responses in COVID-19 patients (who presented obesity (37%) and T2DM (19%)) during the week following the initial diagnosis, where the up-regulated dataset which contains MT2A and MT1-E proteins were positively correlated with M2 in CD8^+^ T cells and these proteins were involved in the cellular response to Zn ion.

Another study about trace elements (iron (Fe), Zn, Cu, and manganese (Mn)) indicated that their deficiency was a risk factor for critical COVID-19 patients. It is worth to note in this study that Zn and Fe levels served as predictors for severity and mortality, respectively. Additionally, it was observed that Cu and MTs levels decreased significantly during the ICU stay of COVID-19 patients, and the changes in MT levels showed an inverse correlation to the changes in albumin levels [187]. Furthermore, elevated albuminuria in COVID-19-positive people with diabetes may potentially be oxidized, inducing cytokine production in the lungs, which could subsequently initiate the cytokine storm [188]. On the other hand, Abouhashem et al. [189] showed that *MT-2A* was downregulated in alveolar type II cells from the elderly in comparison with young patients. This data could be explained by the fact that the elderly are particularly susceptible to Zn deficiency [190]. Therefore, a Zn deficiency does seem to be responsible for the worse outcomes observed in the elderly affected by COVID-19. In addition, it is known that MTs and Zn transporters are expressed in oral tissues that participate in tasting and saliva secretion [191]. A study showed that a dysregulation in the expression of *MTs* associated with COVID-19–induced Zn deficiency might have some effect on oral functions. Therefore, it is expected that COVID-19 patients can improve their oral symptoms with Zn supplementation [192]. Meta-analyses of drugs prescribed worldwide in clinical trials for COVID-19 indicate that the most promising drugs for alleviating COVID-19 symptoms and decreasing the associated mortality are connected to suppressing serum Zn levels and increasing the cellular levels of Zn and MTs [193]. In a recent study, human intestinal tissues biopsied from COVID-19 patients showed that the *MT-1/2* genes are upregulated on lamina propria and epithelial compartments [194]. Additionally, serum ferritin, which is related to Fe metabolism and can be influenced by MTs, is associated with poor outcomes in COVID-19 patients, including those with diabetes [195].

Currently, there is not enough evidence associating MTs with COVID-19 in patients with diabetes and obesity. However, MTs could react to changes in Zn status, which suggests that MTs play a role in the regulation of Zn homeostasis in obesity and diabetes. Thus, it is not surprising that MTs are believed to participate in the regulation of COVID-19 infection.

## Conclusions

Diabetes and obesity are chronic diseases that are becoming increasingly prevalent worldwide. BMI is strongly associated with both diabetes and insulin resistance, highlighting the importance of maintaining a healthy weight to prevent these conditions. MTs, as potent antioxidants, adaptive proteins, and Zn homeostasis regulators play an important role in the prevention of diabetes development. MTs may have a role in preventing diseases associated with obesity, in part by inhibiting the generation of superoxide and endoplasmic reticulum stress induced by the disease, which can cause significant damage. The development of strategies for upregulating MTs would be of great interest to pharmaceutical and medical applications, highlighting the potential of MTs as therapeutic targets. With the insights provided by this comprehensive review, it may be possible to gain a better understanding of the molecular mechanisms that enable MTs to offer protection against the oxidative stress associated with diabetes and obesity. This could lead to a novel therapeutic strategy for these diseases and their associated complications, including protection for patients with diabetes and obesity related to COVID-19.
